# Digital coding of mechanical stress in a dynamic covalent shape memory polymer network

**DOI:** 10.1038/s41467-018-06420-w

**Published:** 2018-10-01

**Authors:** Guogao Zhang, Wenjun Peng, Jingjun Wu, Qian Zhao, Tao Xie

**Affiliations:** State Key Laboratory of Chemical Engineering, College of Chemical and Biological Engineering, Zhejiang University, Hangzhou, 310027 China

## Abstract

Controlling stresses in materials presents many unusual opportunities for their engineering applications. The potential for current approaches is severely limited by the intrinsic tie between the stress and the geometric shape. Here, we report a material concept that allows stress management in a highly efficient digital manner while decoupling the stress and the geometric shape. This is realized in a dynamic covalent shape memory polymer network, for which the elastic shape memory sets the baseline stress level and maintains the geometric shape while the plasticity enabled by the dynamic bond exchange allows stress tuning. With a digital gray scale photothermal mechanism, any arbitrarily defined stress distribution can be created in a free-standing polymer film. The naturally invisible stresses can be further visualized as mechanical colors under polarized light, revealing its potential for encoding hidden information. Our approach expands the technological potential in many areas for which stresses are relevant.

## Abstract (web summary)

Stresses are often detrimental to the mechanical properties of materials but when well controlled can lead to unusual opportunities in various technological areas. Here the authors report a digital photothermal mechanism enabled laser printing that allows freedom in stress manipulation in dynamic covalent shape memory polymers.

## Introduction

Mechanical stresses are ubiquitous in materials. Although they are often detrimental to mechanical properties, well-controlled stresses can lead to unusual opportunities in various technological areas including flexible electronics^1,2^, four-dimensional (4D) printing^3–5^, microfabrication^6^, and actuators^7–9^. Full cycle manipulation of stresses including its generation, storage, and release can therefore have far-reaching consequences. In principle, any conventional shape memory polymer can perform such tasks. However, the intrinsic tie between the internal stress and its geometric shape implies that it is impossible to introduce complex stresses without affecting its shape^10–15^. This characteristic, while essential for shape memory, limits its practical potential in much broader areas.

In this paper, we report a digital photothermal mechanism enabled by laser printing that allows unparalleled freedom in stress manipulation in a dynamic covalent shape memory polymer network, critically without altering its free-standing geometric shape. The digital photothermal effect permits spatiotemporal stress control via plasticity enabled by dynamic covalent bond exchange^16–25^, whereas the elasticity-based shape memory mechanism ensures its geometric stability regardless of the stress^16–20^. This leads to a two-dimensional (2D) film of any arbitrarily distributed stress which is invisible under regular light but can be visualized under polarized light due to the birefringence. Owing to the rich achievable birefringent colors and their actively controllable nature, we call them invisible mechanical colors which are useful for encoding colored hidden information. Our approach of digital stress manipulation in a free-standing polymer expands the technological potential in areas for which stresses are relevant.

## Results

### Polymer design and stress control mechanism

**Fig. 1:**
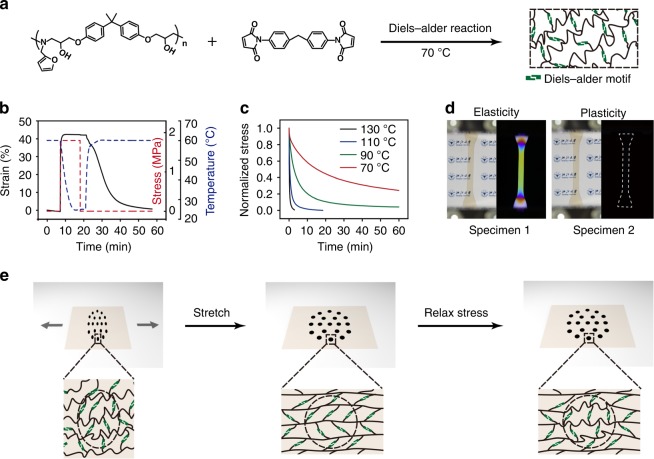
Dynamic covalent shape memory polymer and mechanism for spatially controlled stress. **a** Polymer network synthesis. **b** Quantitative shape memory cycle (black line: strain; red dotted line: stress; blue dotted line: temperature). **c** Plasticity as reflected in stress relaxation at various temperatures. **d** Optical reflection of stresses in two specimens deformed to the same strain (50%) under elastic and plastic deformation mechanisms, respectively (detailed experimental conditions in the Supplementary Methods). The dashed line was drawn to indicate the dimension of Specimen 2. **e** Spatio-selective plasticity in the shape memory network. The changes at the molecular scale are illustrated in the bottom row cartoons with the dashed circle corresponding to the dot

To demonstrate the concept, we use an amorphous shape memory polymer network covalently crosslinked by dynamic Diels–Alder moieties (Fig. 1a) throughout this entire study. Detailed characterization of the polymer network and its precursors are included in the supplementary information (Supplementary Fig. 1 to Fig. 6). This network polymer (gel content: 93.1%) has an onset of glass transition at 42 °C (Supplementary Fig. 1). The maximum strain of this network polymer at 60 °C is 190% (Supplementary Fig. 7), a significant improvement over our previous system due to the reduced crosslinking density^19^. When deformed at 60 °C, the exchange reaction between the Diels–Alder moieties is sufficiently quenched as indicated by its very slow stress relaxation (Supplementary Fig. 8). At this temperature, the material is capable of temporary shape fixing, showing the classical elasticity-based shape memory behavior with shape fixity and shape recovery ratios of 99.4% and 96.7%, respectively (Fig. 1b). At 70 °C or above, the Diels–Alder moieties are activated to undergo more significant bond exchange, leading to faster stress relaxation with higher temperature promoting faster relaxation (Fig. 1c) due to the accelerated Diels–Alder bond exchange. This stress relaxation behavior allows permanent shape reconfiguration via solid-state plasticity^16–19^, opposite to the temporary shape fixing in Fig. 1b. These two deformation modes allow stress to be either temporarily stored or permanently erased. Thus, two identical specimens deformed by the same strain under these two conditions (Fig. 1d) exhibit two completely different stress states: stress fully stored and fully released. These two specimens look identical and transparent under natural light. Under polarized light (dark field unless otherwise noted), however, Specimen 2 completely merges into the black background, while Specimen 1 becomes colored because of its different stress states. Combining these two mechanisms in a spatio-selective manner should therefore allow manipulating stress and its distribution within one sample. Figure 1e illustrates a polymer film with a dot array as an exemplary pattern. Upon stretching, the film is uniformly deformed at both macroscopic and molecular scales. If the stress is relaxed only on the dotted areas via plasticity, this would lead to spatio-selective chain relaxation and hence the spatial difference in stress (Fig. 1e). Importantly, the shape memory function allows any stress field to be locked in the film. Without this function, the non-uniform stress would alter the geometric shape^4–9^ unless the film is anchored onto a substrate^26^. While releasing the non-uniform stress serves its own intended purposes^4–9^, we demonstrate below that decoupling the geometric shape and the stress pattern on a free-standing film has its own unique merits.

### Stress/color manipulation via elastic shape memory effect

**Fig. 2:**
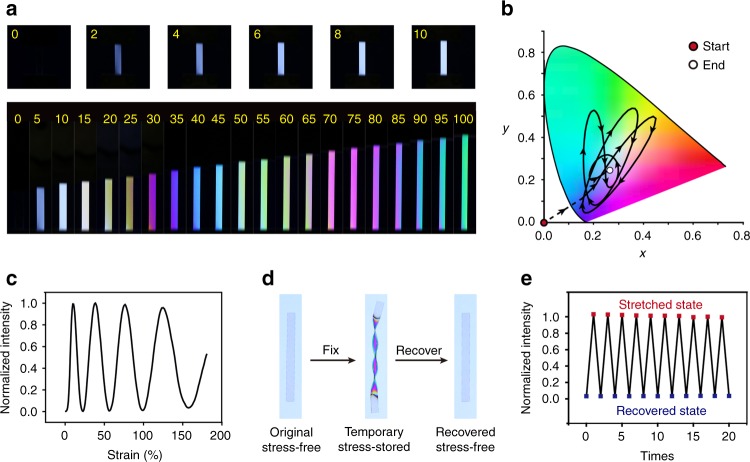
Stress and mechanical color via elastic deformation at 60 °C. **a** Color change during uniaxial stretching at a strain rate of 50% min^−1^ (numbers in yellow denoting strains in percentage). **b** Color evolution during continuous stretching from 0 to 180%, presented at a standard CIE-1931 color space. **c** Cyclic change of the light intensity (450 nm) corresponding to **b**. **d** Photographs (under bright field) of a sample underwent a shape memory cycle (sample length: 4 cm). **e** Light intensity change upon cyclic shape memory experiments (10% strain, 450 nm)

To enable the complex stress manipulation in Fig. 1e, we next investigate independently how elasticity and plasticity affect the stress using mechanical colors as the indicator. To ensure elastic deformation, all the experiments in Fig. 2 were conducted at 60 °C, a temperature sufficiently low to avoid stress relaxation within the relevant experimental timescale. Figure 2a shows the color evolution during uniaxial stretching. At strains below 10%, the color starts at black and gradually transitions into white. At a wider strain range up to 100%, diverse colors are obtained. This color evolution upon stretching is captured in Supplementary Movie 1. The corresponding chromaticity diagram (Fig. 2b) illustrates quantitatively the range of obtainable colors. At a fixed wavelength, the light intensity undergoes a cyclic change (Fig. 2c) upon continuous stretching, with each cycle occurring in an increasingly larger span of strain. The imposed stress/strain, and consequently the color, can be fixed in a temporary shape upon cooling to room temperature and subsequently recover to the stress-free state upon reheating (Fig. 2d). The images in Fig. 2d further demonstrate that, with shape memory function alone, the stress, shape, and color are intrinsically linked together. Owing to the robustness of the shape memory effect, the stress and color can be repeatedly imposed and removed as evidenced by the cyclic change of light intensity upon consecutive shape memory cycles (Fig. 2e).

### Stress/color manipulation via combined elasticity and plasticity

**Fig. 3:**
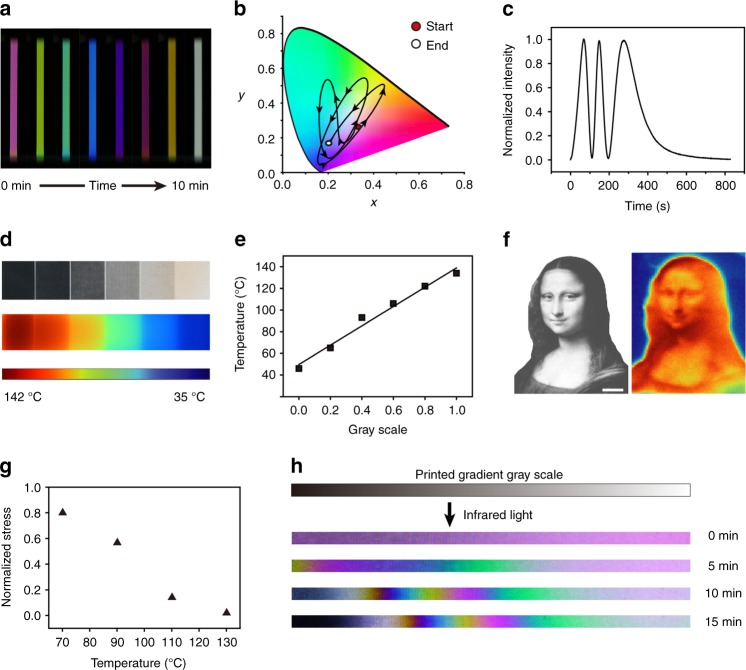
Spatiotemporal control of partial plasticity by digital gray scale photothermal effect. **a** Time evolution of color during stress relaxation at 100 °C (strain: 70%, sample length: 4 cm). **b** Color change presented at a standard CIE-1931 color space (100 °C, strain: 150%). **c** Cyclic variation of light intensity (450 nm) with time corresponding to **b**. **d** Laser printed gray scale pattern (each square is 1 cm × 1 cm, gray scale of 1, 0.8, 0.6, 0.4, 0.2, and 0), and the corresponding infrared thermal image (the third colored line on the bottom represents the temperature scale). **e** Linear correlation between photothermal heating and the gray scale. **f** Gray scale photo of Mona Lisa and its infrared thermal image (the scale bar is 1 cm). **g** Normalized residual stress after photothermal heating for 1 min at different temperatures. **h** Infrared light-induced time evolution of color under polarized light in a pre-stretched film (strain: 70%) printed with a gradient gray scale pattern. Ink is kept on the sample due to the continuous nature of this experiment

Plasticity offers another mechanism to manipulate the stress and color. Whereas complete stress relaxation turns the color into black (Fig. 1d), we suspect that a diverse set of colors can be obtained if a film is allowed to relax to various degrees (partial plasticity), instead of complete relaxation. Indeed, Fig. 3a shows that the color does evolve with time upon stress relaxation at a fixed strain. The obtainable color is diverse as illustrated quantitatively in Fig. 3b. The time evolution of different colors is captured in Supplementary Movie 2. The light intensity at 450 nm undergoes a cyclic change (Fig. 3c) with stress relaxation time, which is different from Fig. 2c in which the color change is induced by strain.

To combine the elasticity and partial plasticity for stress control outlined in Fig. 1e, an additional mechanism that permits spatiotemporal control of partial plasticity is required. We resort to a digital gray scale photothermal effect illustrated in Fig. 3d. We first laser print features of different grayness onto a polymer film. With the exposure of an infrared flood light, the grayness determines the amount of absorbed light, and consequently the temperature distribution (Fig. 3d). The temperature is linearly dependent on the grayness (Fig. 3e), ensuring simplicity in temperature control via printing. Given the flexibility of the laser printing, the temperature distribution can be digitally defined as illustrated through the comparison of a printed image and the corresponding thermal image (Fig. 3f). Due to the strong temperature dependence of stress relaxation kinetics (Fig. 1c), for a fixed relaxation time of 1 min, temperature can determine the degree of partial plasticity (Fig. 3g). Therefore, for a gradient gray scale pattern (Fig. 3h), the corresponding difference in temperature due to the photothermal heating is such that a range of colors can appear and evolve as the partial plasticity progresses. We note that the birefringence color arises from the well-known photoelasticity that is widely used in characterizing mechanical stresses in materials, which are typically passive and detrimental. Not to cause confusion, we emphasize that such photoelasticity (an optical phenomenon) is different from the photothermally induced mechanical elasticity behind the shape memory described in this work. In addition, while mechanical stresses in materials are typically undesirable, the actively controllable nature of the stress enabled by our approach is such that it can be used in a highly constructive manner. For this reason, we call the well-controlled birefringence color as invisible mechanical color.

At a first glance, elastic shape memory and partial plasticity lead to the equivalent result of stress/color manipulation. A critical difference lies in that partial plasticity allows decoupling between the stress and strain/shape, that is, a sample stretched to the same strain can have very different stress states/colors (Supplementary Movie 2). In contrast, stress is intrinsically tied to the strain/shape for elastic deformation (Supplementary Movie 1). Despite the difference, their synergistic roles are key to create and lock any arbitrary stress field in a free-standing film, in terms of both the stress level and its spatial distribution.

### Digital spatio-control of stress

**Fig. 4:**
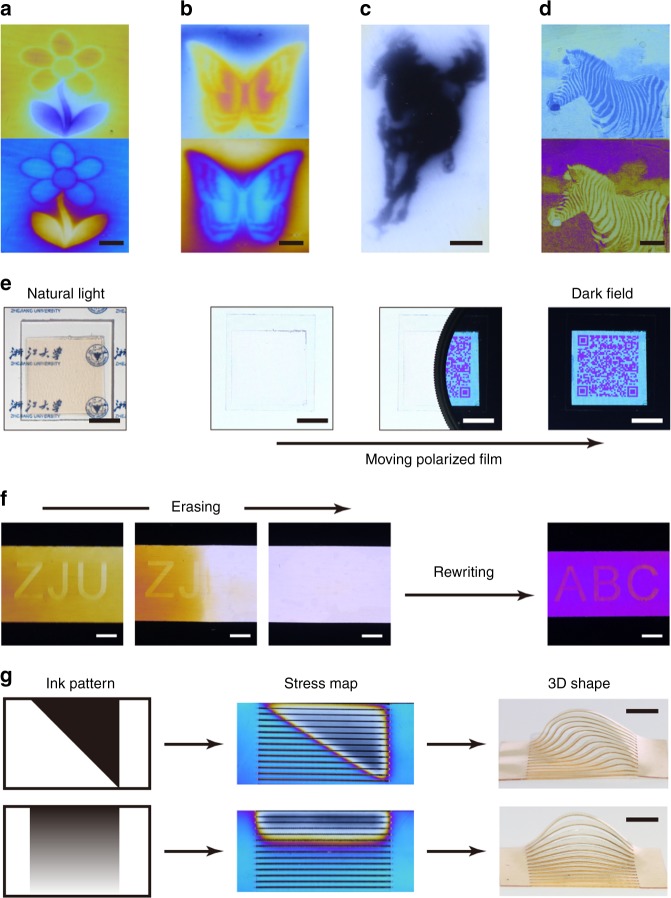
Stress-induced mechanically colored images. **a**, **b** Flower and butterfly painting in bright (upper) and dark field (lower). **c** Horse painting in Chinese traditional style. **d** Images of zebra created via laser direct writing in bright (upper) and dark field (lower). **e** Invisible quick response code. **f** Erasing and rewriting letters. **g** Controlled formation of three-dimensional shapes via heating-induced release (60 °C) of digital stress patterns in two-dimensional free-standing films (black lines in the stress maps represent laser-cut through patterns). All the scale bars are 5 mm

Taking advantages of the flexibility of digital printing, any arbitrary stress field can be coded into a polymer film using the above principles. Briefly, the film is first laser printed with an intended gray scale pattern. The film is then stretched uniaxially to a target strain that is predetermined based on the level of intended background stress/color. However, under the stretching force, the film is exposed to infrared flood light which triggers the spatiotemporal partial plasticity. Subsequently, the film is cooled down to ambient temperature. Upon removing the stretching force, the stress field is locked into the planar film via the shape memory function. To reflect the versatility of the stress control, various mechanically colored images that are all invisible under natural light are created. The flower (Fig. 4a) and butterfly (Fig. 4b) demonstrate the level of control on color. In contrast, the black and white horse (Fig. 4c) extends the scope of the achievable colors, with the different grayness being a key characteristic of Chinese traditional painting. Importantly, our approach yields reproducible color effects (Supplementary Fig. 9). Its spatial resolution is around 200 μm (Supplementary Fig. 10), limited by the resolution of the laser printer and in-plane thermal diffusion of the polymer.

The localized partial plasticity above is achieved by controlling ink printing, and an alternative approach is to control the light. To do so, a polymer film is uniformly printed in black (gray scale of 1). The film is then subject to the same procedure above except that, instead of flood infrared light, laser direct writing is used to trigger the localized photothermal effect. Using this procedure, mechanically colored images of a zebra (Fig. 4d) were generated. Figure 4e shows a quick response code created in this manner. While the encoded stress features are invisible in natural light, they appear as the polarized film moves in (Fig. 4e). The resolution is sufficiently high to ensure its full function, evidenced by successful cell phone scanning of this invisible quick response code under polarized light (Supplementary Movie 3). To further reflect the versatility, Fig. 4f shows that white “ZJU” letters in a yellowish background can be progressively erased by heating to 100 °C under stretching for 3 min. Letters of “ABC” can then be rewritten into the film with an entirely different purple background. This is only possible due to its free-standing nature, which allows the background color to be altered by stretching to a different strain. Besides the color effect, releasing the stress via heating can turn a 2D film into a three-dimensional (3D) shape. Indeed, Fig. 4g shows that different stress patterns can be digitally introduced into two identical films (stretched 40%). An identical through-cut pattern is subsequently generated via laser in its cutting mode. Upon heating above the glass transition temperature, two distinctively different three-dimensional shapes are formed. This convenience and versatility stand in sharp contrast to currently known methods that require complex manual manipulation to alter the permanent shape of a crosslinked polymer^16,18,19^.

The above results are obtained by leveraging a digital photothermal effect. We expect that direct photo-induced plasticity^9^ when combined with the shape memory function should yield similar results. The benefits of using the digital photothermal effect are: more accessible material design choices since many thermally triggered dynamic chemistries are known^27^ compared to rather limited photo-dynamic chemistries; easier control of the light using the combination of gray scale printing and common flood light; broader choice of light wavelength. In contrast, photo-induced plasticity will require highly specialized equipment to produce digital light due to the wavelength limitation. The drawback of the photothermal-induced plasticity is that the resolution is intrinsically limited by thermal diffusion.

## Discussion

In summary, we illustrate that any arbitrary stress distribution can be programmed into a dynamically crosslinked shape memory polymer using mask free digital printing. With the shape memory function setting the background stress, partial plasticity permits spatio-selective and continuous stress manipulation. The material behavior can be expanded to a diverse set of materials owing to recent progresses on dynamic covalent polymer networks^27^. This ability to digitally define stress in a simple way can impact many areas beyond mechanical color. Currently known 4D printing based on stress-induced 2D to 3D transformation^3–5^ all relies on creating non-uniform stress during a liquid to solid transition, whereas this work generates stress patterns in the solid state which will open the door for all solid 4D printing. Manipulating stress is also the key for flexible electronics which typically employ fabrication methods that require planar substrates^1,2^; our material concept can therefore become an interesting enabler. The spatial resolution can be significantly improved by employing higher resolution printing or direct digital light. This, combined with the contact free nature of the light control, makes it attractive for data storage^28^. Our stress control method can also be readily expanded to other stimuli-responsive materials such as liquid crystalline elastomers^29,30^ to further enrich their versatility. Overall, we believe that many more technological opportunities exist beyond those envisioned here.

## Methods

### Polymer synthesis

2,2-Bis(4-glycidyloxyphenyl)-propane (21.1 g, from Sigma-Aldrich) and furfurylamine (6.0 g, from TCI) were dissolved in *N*’*N*-dimethylformamide (DMF, 66.0 g) and reacted at 120 °C for 12 h to yield the furan contained linear polymer solution (*M*_n_ = 10,700 by gel permeation chromatography). Another 24.0 g DMF was added into the solution, and then 1,1’-(methylenedi-4,1-phenylene)bismaleimide (2.3 g, from Sigma-Aldrich) was fully dissolved in the linear polymer solution. Then, 5 mL of the obtained solution was poured into an aluminum dish (90 mm in diameter), and the dish was placed in a 70 °C oven for 5 h to evaporate the solvent. It was further dried in a vacuum oven (70 °C, 48 h) to yield a polymer film with thickness of 0.14 mm upon demolding. The samples were cut into the desired dimensions for various testing. Specifically, polymer films in dogbone shapes (neck dimension of 4 mm × 20 mm) were used in Fig. 1d (specimen 1 and 2) and Supplementary Figure 7. Rectangular specimens (4 mm × 40 mm) were used for Figs. 2d and 3a.

### Stretching experiments

All the uniaxial stretching experiments were carried out using a universal material testing machine (Zwick/Roell Z005) and the strain rate was fixed at 50% min^−1^.

### Photothermal-induced stress relaxation

Patterns were first printed onto the films using a laser printer (HP Color Laser Jet Pro M252) which were then cut into rectangular shapes. All gray scale patterns were created by CoreDRAW X6 software, which also defines quantitatively the grayness. Uniaxial stretching was conducted at 60 °C and the sample was cooled to 25 °C. The film was then removed from the machine and glued onto a glass slide using a double-sided tape. Stress relaxation was conducted by either infrared light exposure or a laser direct writer. The infrared light was provided by a xenon lamp equipped with an infrared filter (CEL-PE300L-3A, Ceaulight company) and the light intensity was 290 mW cm^−2^. The laser direct writer was Speedy 100 R by Trotec company (10640 nm, 25 W) with a maximum resolution of 1000 dpi and the settings were: engrave mode, power 2, speed 2, PPI 1000 Hz. For laser cutting shown in Fig. 4g, the laser writer was set in the cutting mode: power 20, speed 3. The film can be detached from the glass slide upon completion of the stress relaxation and cooling to ambient temperature.

### Optical characterization

Unless otherwise noted, ink was removed by hexane wiping prior to the measurements. All thermal images were obtained with a Fluke Ti10 thermal imager. All polarized optical images were taken using a Canon EOS 70D camera (ISO 1250, shutter speed 1/125, aperture f/4) equipped with a polarizer. A white light-emitting diode (LED) screen was used as the polarized background light. Samples were typically placed between the LED screen and the polarizer in a direction that inclined to the polarization direction of the background light at an angle of 45°. Color spectra were collected using an optical spectrometer (USB2000 UV-VIS, Ocean Optics company). Light intensity at any given wavelength within the measurement range can be extracted from a spectrum. Collecting multiple color spectra during the course of sample stretching or iso-strain stress relaxation allowed monitoring the change in light intensity for a given wavelength.

## Electronic supplementary material

Supplementary Information

Description of Additional Supplementary Files

Supplementary Movie 1

Supplementary Movie 2

Supplementary Movie 3

## Data Availability

The data that support the findings of this study are available from the corresponding author on reasonable request.
