# Supplementary material for: Digital coding of mechanical stress in a dynamic covalent shape memory polymer network
Source: Nat Commun. 2018 Oct 1;9:4002. doi: 10.1038/s41467-018-06420-w (PMC6167378; doi:10.1038/s41467-018-06420-w)
Supplement: Supplementary file 1 — Supplementary Information [file 41467_2018_6420_MOESM1_ESM.pdf]

Supplementary Information

Digital coding of mechanical stress in a dynamic covalent shape memory polymer network

Zhang et al.

Supplementary Methods

Dynamic mechanical analysis (DMA) was conducted with a TA Q800 instrument in "Multi-frequency-strain" mode (frequency 1Hz, heating rate 5 °C min^{-1}). Shape memory tests were conducted in a "Control Force" mode. Stress relaxation were conducted in a "Stress relaxation" mode. Shape fixity and recovery were determined by Equation 1, where ε_{load} represents the maximum strain under load, ε_{rec} is the recovered strain, and ε_d is the fixed strain after cooling and stress removal.

$$R_f = \frac{\varepsilon_d}{\varepsilon_{load}} \text{ and } R_r = \frac{\varepsilon_d - \varepsilon_{rec}}{\varepsilon_d} \quad (1)$$

Gel permeation chromatography (GPC) was conducted with a Waters 1525 instrument with a refractometric detector. The solvent was tetrahydrofuran (THF) and the calibration was done with monodisperse polystyrene.

Differential scanning calorimetry (DSC) was conducted in a TA Q200 instrument. The test procedures are as follow: 1. Equilibrate at 70 °C; 2. Isothermal for 10 min; 3. Equilibrate at -20 °C; 4. Isothermal for 5 min; 5. Ramp 10 °C min^{-1} to 200 °C. Data were recorded for the second heating process (-20 °C to 200 °C).

Fourier transform infrared (FTIR) spectra were recorded with a ThermoFisher Nicolet5700 Fourier transformation infrared spectrometer. Furan-containing linear polymer solution was coated on the surface of a KBr tablet, and FTIR spectrum was recorded after the solvent evaporation. FTIR spectra of the mixture of the furan-containing linear polymer and BM were collected before and after a heat treatment at 70 °C for 1 h.

Thermal gravimetric analysis (TGA) was carried out with a TA Q500 Instrument under nitrogen atmosphere. Sample was heated from 0 °C to 700 °C at a heating rate of 10 °C min^{-1}.

Gel content test: 96.2 mg of the sample was soaked in dimethylformamide (100 mL) for three days with the solvent being refreshed daily. Afterwards, the residual polymer sample was dried at 70 °C in a vacuum oven until the weight reached a constant value (89.6 mg). The gel content was 93.1%, determined as the weight ratio of the polymer after and before the solvent extraction.

Experimental conditions for Figure 1d in the main text: Specimen 1 was equilibrated at 60 °C for 5 min and stretched by 50%. Upon cooling down in natural air, the photo was taken. Specimen 2 was equilibrated at 130 °C for 5 min. It was then stretched by 50% and maintained isothermal for 5 min. The photo was taken after the specimen was cooled down in natural air.

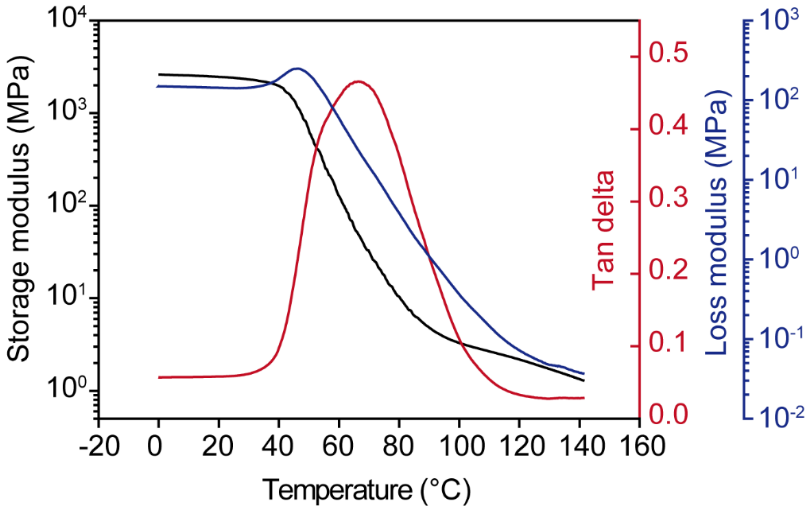

Supplementary Figure 1. DMA curves show an onset of glass transition at 42 °C (Multi-frequency-strain mode, 1 Hz, 5 °C min^{-1}).

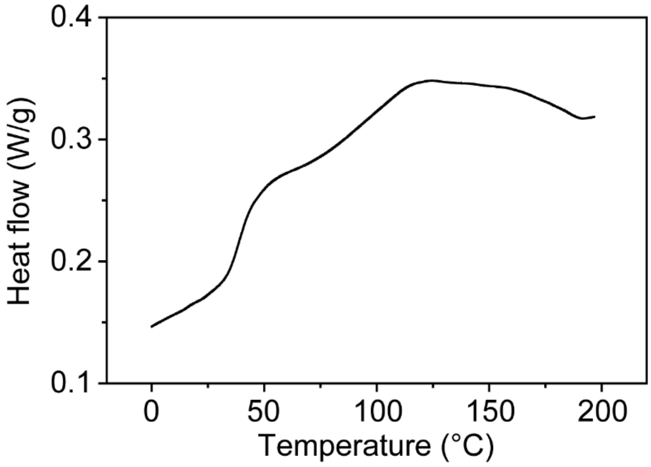

Supplementary Figure 2. The first endothermic peak shows a glass transition at 38 °C. The second endothermic peak corresponds to the retro-DA reaction at elevated temperature.

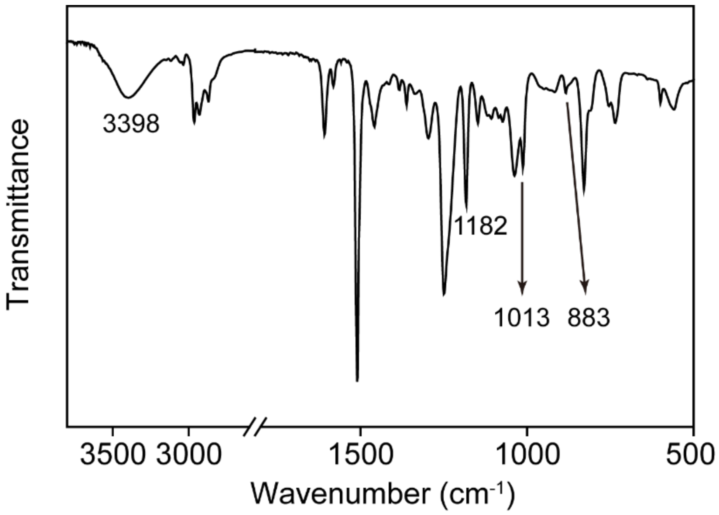

Supplementary Figure 3. FTIR spectrum of the furan-containing linear polymer. Peaks at 3398 cm^{-1} and 1182 cm^{-1} are characteristic to hydroxyl groups, confirming the occurrence of the ring-opening reaction between amine and epoxy groups. Other characteristic peaks confirming the structures are: furfuryl ring (1013, 883 cm^{-1})[1].

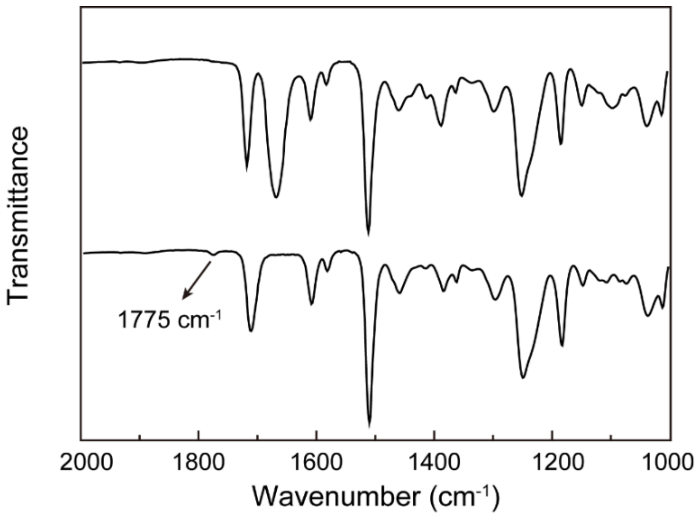

Supplementary Figure 4. FTIR spectra of a mixture of the furan-containing linear polymer and BM (top), and the mixture treated at 70 °C for 1 h (below). The peak at 1775 cm^{-1} is specific to DA adducts of maleimide and furan[2].

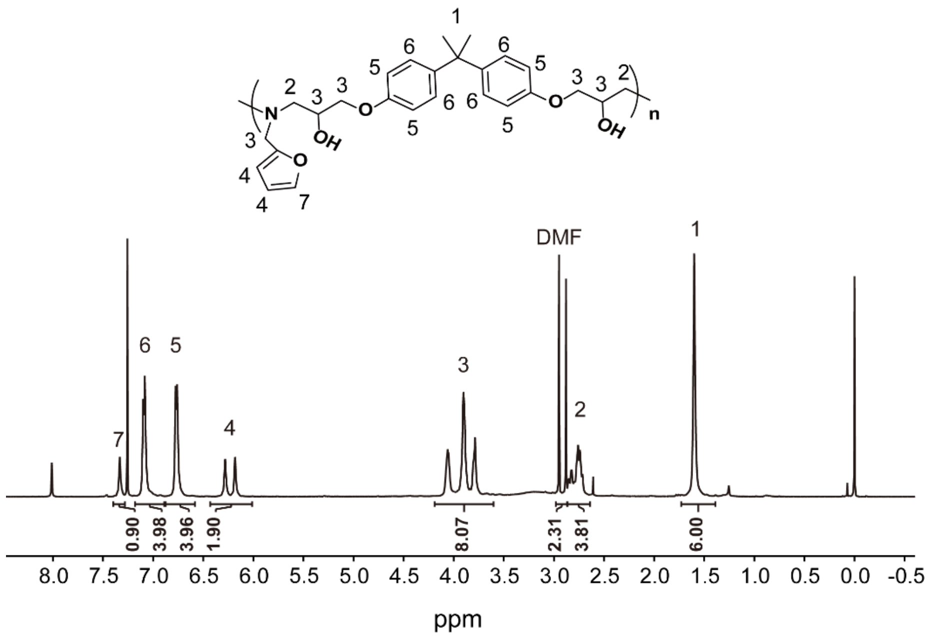

Supplementary Figure 5. NMR spectra of the furan-containing linear polymer.

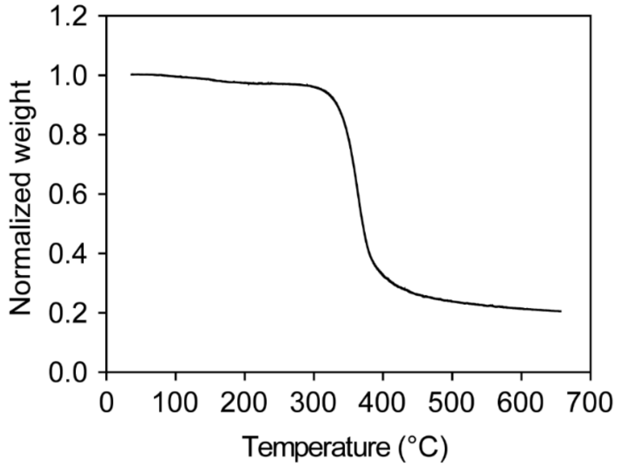

Supplementary Figure 6. TGA curve under nitrogen atmosphere (0 to 700 °C, 10 °C min^{-1}).

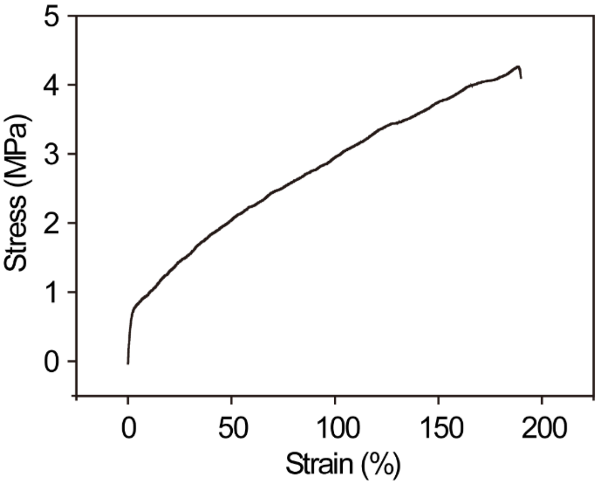

Supplementary Figure 7. Stress-strain curve at 60 °C obtained using a Zwick/Roell Z005 machine.

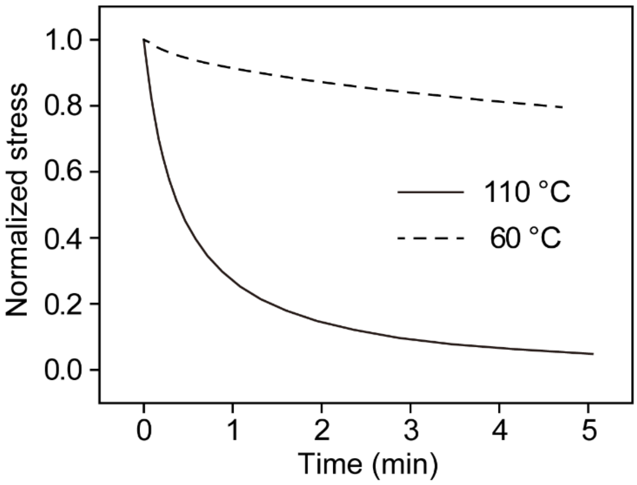

Supplementary Figure 8. Stress relaxation curves at 110 °C and 60 °C. The much slower stress relaxation at 60 °C is due to the combined effect of the intrinsically slower exchange reaction between the Diels-Alder moieties and the freezing of the molecular mobility owing to the overlap with the glass transition (Supplementary Figs S1 and 2)

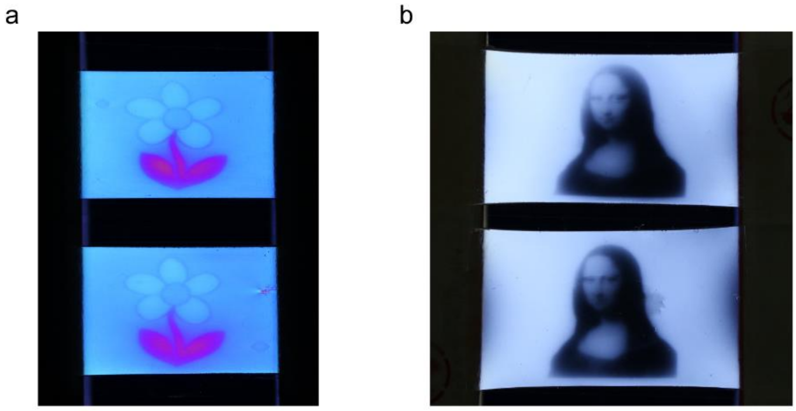

Supplementary Figure 9. Stress control repeatability experiments (observed in dark field). (a) Two parallel samples printed with an identical flower pattern were pre-stretched to 40% at 60 °C, and followed by exposure to infrared light for 30 s. (b) Two parallel samples printed with Mona Lisa were pre-stretched to 10%, and followed by exposure to infrared light for 60 s. The minor defects in the bottom two images originate from the original films prior to the digital stress coding, they serve as important distinction between otherwise identical images.

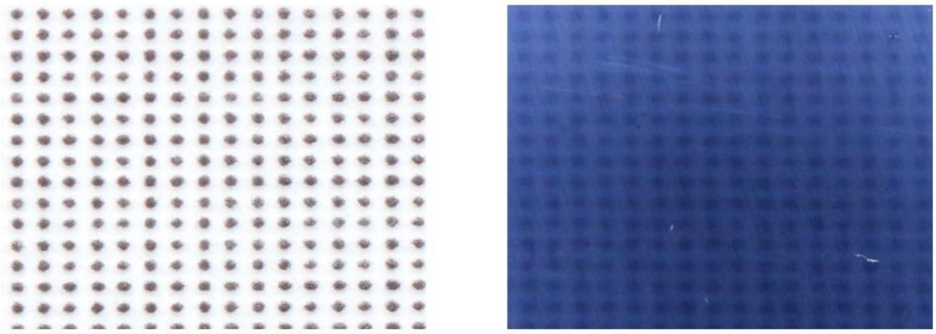

Supplementary Figure 10. Optical image of an as-printed ink pattern (arrays with diameter of 200 μm) (left) and the corresponding polarized optical image of stress pattern after the ink was removed (right). Arrays with diameter of 100 μm becomes too blurred to distinguish in the stress pattern.

Supplementary References

1. Liu, Y. L., & Hsieh, C. Y. *J. Polym. Sci. Pol. Chem.* **44**, 905-913 (2006).
2. Tian, Q., Yuan, Y. C., Rong, M. Z. & Zhang, M. Q. A thermally remendable epoxy resin. *J. Mater. Chem.* **19**, 1289-1296 (2009).
