# Supplementary material for: Digital coding of mechanical stress in a dynamic covalent shape memory polymer network
Source: Nat Commun. 2018 Oct 1;9:4002. doi: 10.1038/s41467-018-06420-w (PMC6167378; doi:10.1038/s41467-018-06420-w)
Supplement: Supplementary file 2 — Description of Additional Supplementary Files [file 41467_2018_6420_MOESM2_ESM.pdf]

Description of Additional Supplementary Files

File Name: Supplementary Movie 1
Description: Evolution of mechanical color in elastic stretching

File Name: Supplementary Movie 2
Description: Evolution of mechanical color in plastic stress relaxation

File Name: Supplementary Movie 3
Description: Scanning of a mechanical colored QR code
